# Brain Correlates of Persistent Postural-Perceptual Dizziness: A Review of Neuroimaging Studies

**DOI:** 10.3390/jcm10184274

**Published:** 2021-09-21

**Authors:** Iole Indovina, Luca Passamonti, Viviana Mucci, Giuseppe Chiarella, Francesco Lacquaniti, Jeffrey P. Staab

**Affiliations:** 1 Department of Biomedical and Dental Sciences and Morphofunctional Imaging, University of Messina, 98125 Messina, Italy; 2 Laboratory of Neuromotor Physiology, IRCCS Santa Lucia Foundation, 00179 Rome, Italy; viviana.mucci@gmail.com (V.M.); lacquaniti@med.uniroma2.it (F.L.); 3 Department of Clinical Neurosciences, University of Cambridge, Cambridge CB2 0QQ, UK; passamontiluca@gmail.com; 4 Institute of Bioimaging & Molecular Physiology, National Research Council, 20054 Milano, Italy; 5 School of Science, Western Sydney University, Sydney, NSW 2000, Australia; 6 Unit of Audiology, Department of Experimental and Clinical Medicine, Magna Græcia University, 88100 Catanzaro, Italy; chiarella@unicz.it; 7 Department of Systems Medicine and Centre of Space BioMedicine, University of Rome Tor Vergata, 00133 Rome, Italy; 8 Departments of Psychiatry and Psychology and Otorhinolaryngology—Head and Neck Surgery, Mayo Clinic, Rochester, MN 55905, USA

**Keywords:** persistent postural-perceptual dizziness, phobic postural vertigo, chronic subjective dizziness, visually induced dizziness, visual dependency, neuroimaging

## Abstract

Persistent postural-perceptual dizziness (PPPD), defined in 2017, is a vestibular disorder characterized by chronic dizziness that is exacerbated by upright posture and exposure to complex visual stimuli. This review focused on recent neuroimaging studies that explored the pathophysiological mechanisms underlying PPPD and three conditions that predated it. The emerging picture is that local activity and functional connectivity in multimodal vestibular cortical areas are decreased in PPPD, which is potentially related to structural abnormalities (e.g., reductions in cortical folding and grey-matter volume). Additionally, connectivity between the prefrontal cortex, which regulates attentional and emotional responses, and primary visual and motor regions appears to be increased in PPPD. These results complement physiological and psychological data identifying hypervigilant postural control and visual dependence in patients with PPPD, supporting the hypothesis that PPPD arises from shifts in interactions among visuo-vestibular, sensorimotor, and emotional networks that overweigh visual over vestibular inputs and increase the effects of anxiety-related mechanisms on locomotor control and spatial orientation.

## 1. Introduction

Persistent postural-perceptual dizziness (PPPD) is a chronic functional vestibular disorder defined by the Barany Society in 2017 for the International Classification of Vestibular Disorders [1] and included by the World Health Organization in the forthcoming 11th edition of the International Classification of Diseases [2]. The concept of PPPD was drawn from four previously described conditions: two clinical diagnoses, phobic postural vertigo (PPV) [3] and chronic subjective dizziness (CSD) [4], and two complex symptoms, visually induced dizziness (VID) [5] and space and motion discomfort (SMD) [6]. Historically, reports of clinical syndromes resembling PPPD can be traced to the 1870 s, including the first description of “agoraphobia” by the German neurologist Carl Westphal [7], which included space and motion symptoms that no longer exist in definitions of the psychiatric disorder of that name. The clinical hallmark of PPPD is the presence of chronic dizziness, unsteadiness, and swaying or rocking (non-spinning) vertigo that wax and wane throughout the day and last a minimum of 3 months. These core vestibular symptoms are exacerbated by upright posture, active or passive self-motion, and exposure to environments with complex or moving visual stimuli [8,9]. PPPD may be triggered by various conditions that share an ability to produce acute or recurrent bouts of vertigo, unsteadiness or dizziness or disrupt balance function, including peripheral and central vestibular disorders, migraine, anxiety disorders, autonomic disorders, mild traumatic brain injury, metabolic disorders, cardiac dysrhythmias, and adverse reactions to medications [1,10,11]. PPPD superseded CSD. Some investigators retained the diagnosis of PPV to designate patients with clinically significant phobic symptoms in addition to core features of PPPD. VID and SMD remain as complex vestibular symptoms. They are key elements of PPPD, but may occur with or following other peripheral or central vestibular disorders [6].

Since the definition of PPPD was published in 2017, clinical studies investigating its point prevalence found rates of 14% among patients presenting with a chief complaint of chronic dizziness to a general medical practice [12], about 20% among all patients consulting with hospital-based neurologists for vestibular symptoms [13,14,15], and more than 40% among patients attending a subspecialty practice designed specifically for managing individuals with chronic dizziness [16], making it the most common cause of persistent vestibular symptoms in all of these settings.

The current diagnosis of PPPD is based on careful recognition of its core clinical symptoms [1,17] as it has no diagnostic tests or biomarkers [1]. Case series, uncontrolled clinical trials, and recently emerging controlled trials support the use of vestibular rehabilitation, serotonergic antidepressants, and cognitive behavioral psychotherapy, often in combination, to treat PPPD (for reviews see [11,18]).

In terms of pathophysiology, the following specific mechanisms have been proposed to explain the initial development and persistence of PPPD. These hypotheses are based largely on research conducted on patients who had one of the four predecessors of PPPD, but are gaining increasing support from investigations conducted on patients diagnosed with PPPD itself. An anxiety diathesis in the form of anxiety-related personality traits (neuroticism, introversion) [19,20,21] or a pre-existing personal or family history of anxiety disorders [22] is thought to predispose to PPPD. Then, acute anxiety and increased body vigilance regarding the physical sensations of dizziness in response to precipitating events appears to promote its development [23,24,25,26]. These two anxiety-related factors appear to interact with physiologic mechanisms that control locomotion and spatial orientation to induce two more pathophysiological processes that produce the core symptoms of persistent dizziness, unsteadiness, and hypersensitivity to complex or moving visual stimuli. First, heightened vigilance with respect to standing and walking stiffens postural control and alters gait, similar to what has been observed in normal individuals when standing on or traversing elevated or insecure surfaces [27,28,29,30,31]. This may be due to a lower threshold for activating closed-loop feedback control mechanisms that are typically reserved for challenging demands on locomotion [31]. In addition, patients with PPPD and its predecessors were found to have a greater reliance on visual rather than vestibular inputs for determining spatial orientation and maintaining balance (i.e., visual dependency, the physiologic process thought specifically responsible for VID) [23,32,33,34,35,36,37]. The interaction of these psychological and physiological processes in the brain is hypothesized to alter the internal representation of the body in space [38,39] and degrade postural control and locomotion in simple and complex environments [27,40].

Despite the formulation of such pathophysiological models, the neural underpinnings of PPPD remain unclear and poorly investigated. Illuminating the neural basis of PPPD may have important consequences in terms of developing more objective markers for its diagnosis and more reliable approaches its treatment [41]. Different neuroimaging studies of patients with PPPD and predecessor syndromes using functional and structural techniques have begun to shed light on its etiopathogenesis [36,39,42,43,44,45,46,47,48,49,50,51]. Herein, we review recently published studies of structural and functional correlates of PPPD, PPV, CSD, and VID and suggest new avenues for future neuroimaging research the better understand the specific brain mechanisms underlying PPPD.

## 2. Materials and Methods

This review was performed using literature databases, including PubMed, Google Scholar and Scopus, from inception until October 2020. We used the following search terms in different combinations: “Dizziness”, “Imaging”, “Chronic Subjective Dizziness”, “Persistent Postural-Perceptual Dizziness”, “Phobic Postural Vertigo”, “Visually Induced Dizziness”, “Visual Dependence”, “Space Motion Discomfort”, “Functional Near-Infrared Spectroscopy”, “Neuroimaging”, “Magnetic Resonance Imaging (MRI)”, “Structural”, “Functional Connectivity”, “Single Photon Emission Computed Tomography (SPECT)” and “Functional” “Task Based MRI”, “Grey Matter Volume”, “Diffusion Tensor Imaging”, “Diffusion Weighted Imaging”. To compare the results from different studies, within a unified and standard nomenclature, the Montreal Neurological Institute (MNI) coordinates were mapped from functional and structural imaging studies into regions of the cyto-architectonic Anatomy atlas [52], complemented with the connectivity-based Brainnetome atlas [53].

## 3. Results

### 3.1. Comprehensive Screening of PPPD Neuroimaging Data

Six fMRI studies investigated functional alterations in patients diagnosed with PPPD, CSD, PPV, or VID relative to healthy controls using different paradigms, such as sound-evoked vestibular stimulation [39], various visual motion stimuli [48,50], visually evoked self-motion simulation [47,49], and caloric vestibular stimulation [50] (Table 1). Four additional fMRI studies were conducted at ‘rest’ [36,42,43,44]. One study used SPECT to assess the brain metabolic differences between patients with PPPD and healthy controls [45]. Three studies investigated brain structural alterations, one via surface-based morphometry (SBM) and two with voxel-based morphometry (VBM) [46,48,51].

### 3.2. Structural Neuroimaging Studies

#### 3.2.1. Decreases in Structural Neuroimaging Parameters (Grey Matter Volume, Cortical Thickness and Cortical Folding)

**Figure 1:**
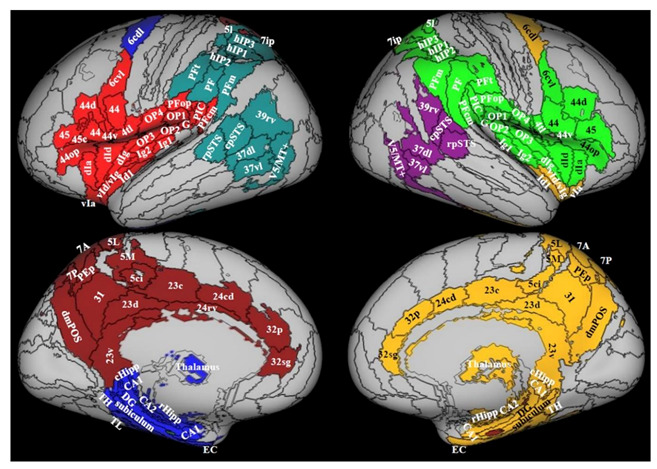
Multimodal vestibular cortex. Reproduced with permission from Indovina et al., 2020 [55], published by Elsevier Inc. Areas in color were selected as belonging to the multimodal vestibular cortex. Each color represents a module, that is, a cluster of areas highly connected. Data from 794 right-handed individuals. There are 4 modules on the left and 3 on the right. Regions from the Anatomy atlas [52] and Brainnetome atlas [53] are overlapped onto the Conte69 inflated brain in workbench viewer [63]. Parietal opercula 1, 2, 3, 4 (OP1, 2, 3, 4), hypergranular insula (G), superior temporal sulcus (STS), visual motion complex (V5/MT+), caudal dorsolateral area 6 (6 cdl), area 4 tongue and larynx region (4 tl), granular insula 2 (Ig2), granular insular 1 (Ig1), Area supramarginalis opercularis (PFop), rostroventral area 40 (PIC), area supramarginalis columnata magnocellularis (posterior) (PFcm), area supramarginalis tenuicorticalis (PFt), area supramarginalis (PF), area supramarginalis magnocellularis (PFm), right posterior superior temporal sulcus (rpSTS), caudal posterior superior temporal sulcus (cpSTS), dorsolateral area 37 (37dl), ventrolateral area 37 (37vl), rostroventral area 39 (39 rv), human intraparietal 1, 2, 3 (hIP1, 2, 3), lateral area 5 (5I), intraparietal area 7 (7ip), superial parietal lobe: 7A, 5L, 7O. Parvicellular superior parietal area (PEp), precuneus (5M), superior parietal lobe (5ci), area 31 (31), dorsomedial parietooccipital sulcus (dmPOS), dorsal area 23 (23d), caudal area 23 (23c), ventral area 23 (23rv), caudodorsal 24 (24cd), pregenual area 32 (32p), subgenual area 32 (32sg), caudal hippocampus (cHipp), cornu ammonis 1 (CA1), hippocampotemporalis (TH), subiculum, latero posterior parahippocampal gyrus (TL), Cornu Ammonis 2 (CA2), dental gyrus (DG), rostal hippocampus (rHipp), cornu ammonis 1 (CA1), entorhinal cortex (EC), caudal ventrolateral area 6 (6cvl), dorsal area 44 (44d), rostral area 45 (45), caudal area 45 (45c), opercular area (44op), dorsal agranular insular (dIa), ventral agranular insula (vIa), ventral dysgranular and granular insula (VId/dIg), dysgranular insula (Id1), dorsal granular insula (dIg), dorsal dysgranular insula (dId), granular insula 2 (Ig2), ventral area 44 (44v).

Three studies investigated structural alterations through surface-based morphometry (SBM) [46] and voxel-based morphometry (VBM) [51] in patients with PPPD, and both analysis techniques in patients with PPV [48] (Table 1). These studies consistently showed that PPPD and its predecessors were associated with decreases in grey matter volume (GMV) [51], GMV and cortical thickness [48], and cortical folding [46] in areas of the multi-modal vestibular network (Figure 1) [55]. This circuit is specialized for processing visual signals related to self-motion and gravity perception [56,57,58,59,60,61,62].

**Figure 2:**
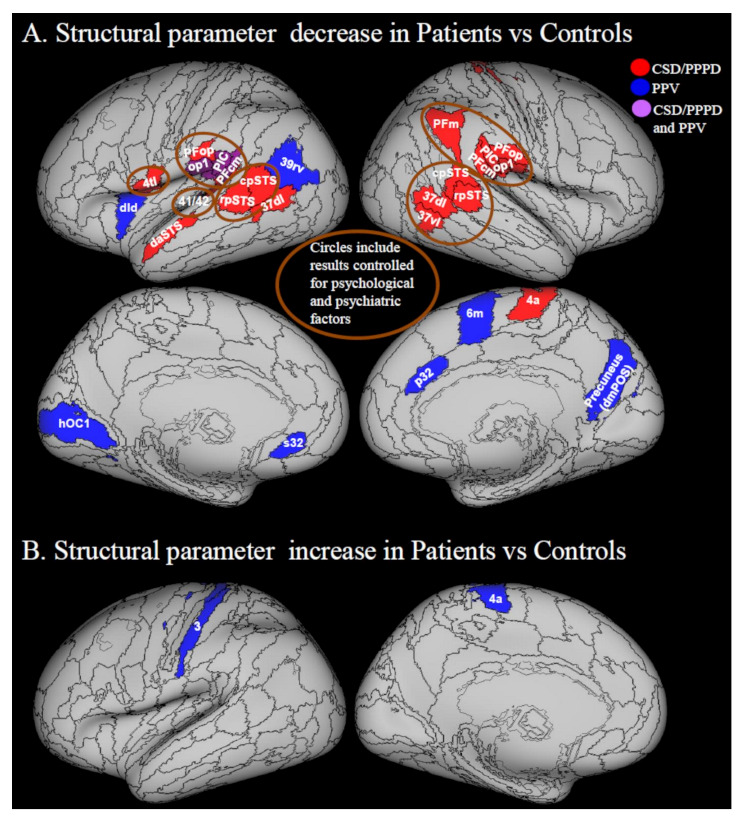
Structural parameters in Patients vs. Controls. Regions from PPPD are in red, regions from PPV are in blue and regions in common are in purple. (**A**) Parameter reduction. Wurthmann et al. [51] found reduced grey matter in patients with PPPD compared to healthy controls in the superior temporal sulcus (STS), left dorsal anterior (daSTS = BA 22r) and left rostral posterior (rpSTS) regions, left middle temporal gyrus in the dorso-lateral Brodmann area (BA) 37 (37 dl), and right primary motor cortex in BA 4 a. Popp et al. [48] found grey matter decrease in the left rostroventral BA 40 (PIC) and left PFcm within the supramarginal gyrus, left parietal operculum OP1 and right medial premotor cortex BA 6 (6m). They also found reduced cortical thickness in the left middle insula (dorsal dysgranular insula = dId), left inferior parietal cortex in the rostro-ventral BA 39 (39rv), left primary visual cortex (hOC1), left ventromedial prefrontal cortex in the subgenual BA 32 (s32), right anterior cingulate cortex in the pregenual BA32 (32p), and in the right precuneus in the dorso-medial parietal occipital sulcus (dmPOS). Nigro et al. [46] found reduced cortical folding in left superior temporal gyrus (BA 41/42), bilateral rostral and caudal posterior superior temporal sulcus (rpSTS, cpSTS), left BA 4 larynx and tongue region (4tl), bilateral suprarmarginal gyrus and adjacent parietal operculum (Pfop, PFcm, PIC, op1), right PFm and right ventro-lateral BA 37 (37 vl, 37dl). Circled areas come from this last analysis and are controlled for psychological and psychiatric variables. (**B**) Parameter increase. Popp et al. found increased grey matter volume in left somato-motor cortex (BA 3, 4a). Regions from the Anatomy atlas [52] and Brainnetome atlas [53] are overlapped onto the Conte69 inflated brain in workbench viewer [63].

GMV and cortical thickness are measures of grey matter quantity, while cortical folding is thought to be a measure of the mechanical tension along the axons that connect different regions [64], and thus a decrease in cortical folding reflects a decrease in white matter connections. Affected areas included posterior perisylvian regions such as the retro-insula (also identified as posterior insular cortex, PIC) [55] and area PFcm within the supramarginal gyrus [65], the parietal operculum (OP1), the middle insula (dorsal dysgranular insula, (dId)), and the superior temporal gyrus (Figure 2A). The PIC is a key region of the cortical vestibular system, responding to both vestibular and visual stimulation [66]. Decrease of grey matter and/or cortical folding was also observed in prefrontal regions including the inferior frontal gyrus (BA 44 pars opercularis (44op)), subgenual/pregenual cingulate areas (s32, p32), premotor cortex (medial BA 6), medial and inferior temporal gyrus (BA 37), and precuneus (see Figure 2A for details). Finally, decrease of GMV was reported in the right primary motor cortex (BA 4a) and reduced cortical thickness was reported in the left primary visual cortex, in the occipital area (hOC1) (see Figure 2A for details).

These imaging studies included patients with both peripheral vestibular (29/95) and non-vestibular (66/95) triggers of PPPD or PPV, meaning that their results may have been confounded by changes related directly to the precipitants of illness rather than to PPPD. A study by zu Eulenburg et al. demonstrated this possibility [67]. They imagined a group of patients who remained symptomatic and had residual canal paresis and abnormal tilt of the subjective visual vertical an average of 2.5 years following onset of vestibular neuritis [67]. Using voxel-based morphometry, they found decreased grey matter volume in the superior frontal gyrus (right BA6 dorsolateral), hippocampus, and superior temporal sulcus (STS) [67], findings that partially overlap the studies of PPPD and PPV [46,48,51]. Unfortunately, zu Eulenburg et al. did not provide full diagnostic assessments of their patients’ chronic symptoms. It is likely that at least some of them had PPPD with or without residual uncompensated peripheral vestibular deficits. Thus, the more recent studies of PPPD and PPV [46,48,51] and the older study of zu Eulenberg et al. [67] have the opposite shortcomings. The former provides formal diagnoses for their patients’ chronic symptoms but include patients with a mix of acute precipitants, whereas the latter includes a single precipitant, but fails to fully characterize patients’ chronic symptoms. Thus, it is not clear if the structural changes found in these studies were due to compensatory mechanisms for inciting peripheral vestibular deficits (more likely in the brainstem) or structural changes that developed secondary to functional alterations associated with PPPD (more likely in the cortex). Future studies will have to compare patients who develop PPPD secondary to specific vestibular triggers with control groups that develop PPPD after other precipitants.

Co-existing depression and anxiety present another potential confound, as some of the structural changes noted in PPPD and PPV could be partially driven by unmatched psychological and psychiatric factors in these patients [48,51]. Anxiety was not measured by Popp et al. in their investigation, but it could have been particularly high, as 15 of 37 patients withdrew from a subsequent functional imaging protocol after structural images were obtained due to high anxiety. Anxiety and depression have been found to affect neural plasticity of limbic and prefrontal cortices [68,69].

Nigro et al. controlled for depression and anxiety when assessing surface morphometric indices in PPPD [46]. Areas from this analysis are circled in Figure 2A. Qualitatively, there is considerable overlap in the posterior peri-sylvian region among results from studies that controlled and studies that did not control for psychiatric and psychological variables, suggesting that some of the functional changes that they reported were due to PPPD or PPV. However, studies that did not control for psychological variables showed more extended and diffuse decrease of indices of grey matter quantity, particularly in the medial region of cerebral hemispheres.

In terms of limitations, all the examined studies were cross-sectional in their design, which makes it difficult to draw firm conclusions on whether the changes observed were a consequence of the disorders investigated or whether they were pre-existing alterations. Some patients were taking antidepressant medications to treat PPPD at the time of neuroimaging [51], which might be another confound to the interpretation of grey matter volumes in limbic and prefrontal areas [70]. Ultimately, prospective studies will be needed to evaluate whether any brain abnormalities might increase the risk of developing PPPD, but those will be difficult and costly to perform until more data are available to identify the patients who are most likely to experience PPPD after its range of potential precipitants. Until then, proper characterization of precipitants, medical and psychiatric comorbidities, and treatments received, as well as enrolment of cohorts large enough to control for these confounds will be necessary design elements, even for cross-sectional investigations. 

#### 3.2.2. Increases in Structural Neuroimaging Parameters

The study by Popp et al. [48] was the only investigation showing increased grey matter volumes in any area of the brain. However, as previously noted, a substantial portion of patient participants in that study had levels of anxiety high enough to cause them to withdraw from the functional imaging portion of the protocol. Thus, the results may be confounded by levels of trait anxiety or the existence of pre-existing anxiety disorders that could have induced cortical plasticity. With that caveat in mind, it is worth noting that Popp et al. found a structural increase in the leg area of the primary motor cortex [48] (Figure 2B). It is known that patients with PPV adopt gait changes that correlate with their fear of falling [28]. Hence, an aberrant use of anti-gravity muscles to maintain the chronically stiffened postural control associated with PPV could be a reason for such changes.

### 3.3. Functional Neuroimaging Studies

#### 3.3.1. Decreases in Local Activity

**Figure 3:**
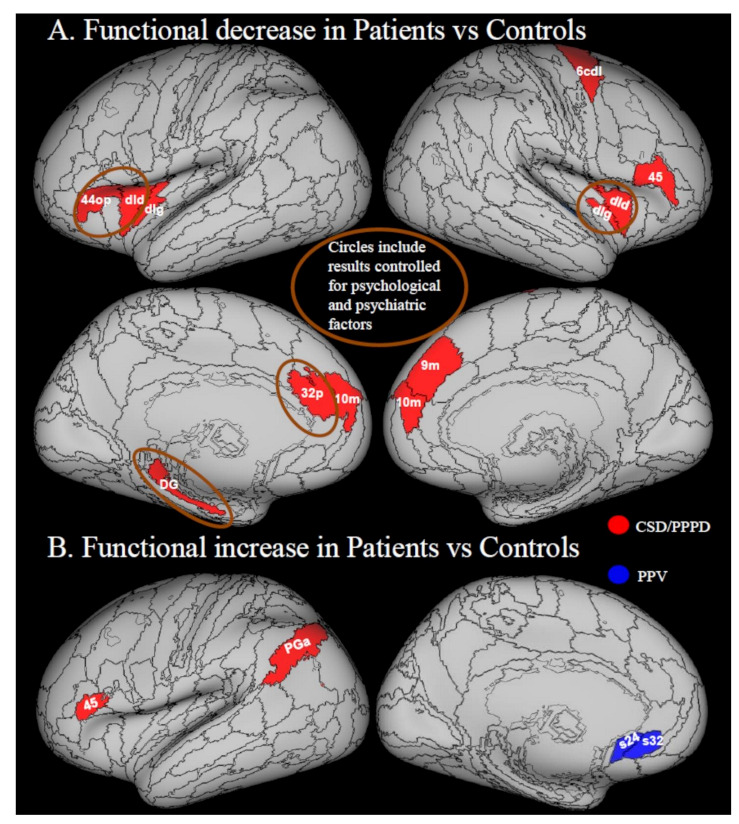
Functional response in Patients vs. Controls. Regions from PPPD are in red, regions from PPV are in blue. (**A**) Functional parameter reduction. Indovina et al. [39] found decreased response to sound-evoked vestibular stimulation in the left hippocampus (dentate gyrus = DG), left inferior frontal gyrus (opercular BA 44 = 44 op)/anterior insula, right posterior insula (dorsal granular insula = dIg) and left pregenual cingulate cortex (32 p). Riccelli et al. [49] found decreased response to vertical vs. horizontal visual motion in right middle insula (dId). Na et al. [45] found decreased response during rest in right BA 45, right BA 6 cdl, left dIg, bilateral BA10 m, left FO2 (not visible), right BA 9m. (**B**) Functional parameters increase. Popp et al. [48] found increased response in the subgenual cingulate cortex (s24, s32) in patients with PPV both during visual motion stimulation and motion aftereffect. von Söhsten Lins et al. [54] found increased response in the left PGa angular gyrus in patients with PPPD in response to negative vs. positive pictures. Passamonti et al. [47] considered the effect of neuroticism after matching the control group for neuroticism and found increased response in neurotic patients in response to vertical vs. horizontal stimulation in left BA45. Regions from the Anatomy atlas [52] and Brainnetome atlas [53] are overlapped onto the Conte69 inflated brain in workbench viewer [63].

Patients with PPPD or its predecessors showed a decrease in the local activity in areas belonging to the multi-modal vestibular cortex compared to healthy controls during sound-evoked vestibular stimulation [39], visual motion stimulation [49] and resting state fMRI [45]. Prefrontal regions, insula and hippocampus showed decreased activity during sound-evoked vestibular stimuli in patients with CSD [39] or resting state in patients with PPPD [45] (Figure 3A). More specifically, patients with CSD were assessed during auditory stimulation with loud short tone bursts (STB), which are known to evoke a vestibular response, relative to soft STB and white noise, which evoke only an auditory response. The contrast of these stimuli is known to evoke local responses in the vestibular cortex while controlling for the sound evoked activations in the primary auditory cortices [39]. Thus, the vestibular stimulation resulting after subtraction of the auditory component is used to assess brain activity and potential anomalies in this activation. In patients with CSD, relative to controls, such sound-evoked vestibular brain activity was significantly reduced in the left inferior frontal gyrus (44 op)/anterior insula, left hippocampus (dentate gyrus, DG), left pregenual cingulate cortex (p32) and right posterior insula (dIg) (Figure 3A), thus suggesting a general alteration of the activity of multimodal vestibular cortex core regions [39]. Riccelli et al. [49] studied fMRI response in patients with CSD vs. healthy controls during visual simulation of virtual rollercoaster rides, a stimulus exacerbating patients’ symptoms [1]. A decreased response was detected in the right middle insula (dId) in patients with CSD vs. healthy controls during rides along vertical vs. horizontal portions of the rides (Figure 3A) [49]. This result may have been related to a fear-inducing perception of instability during the vertical vs. horizontal condition. However, heart rate acquired during fMRI acquisition did not show differences between the two conditions thus indirectly suggesting that the vertical condition was not perceived as more threatening. Instead, it was hypothesized that the difference between the vertical and horizontal condition might be related to altered coding of the internal model of gravity [58]. In particular, it was hypothesized that over-reliance on visual information might reduce the use of the internal model of gravity to predict the sensory consequences of motion. Relying on visual feedback rather than predictive mechanisms could lead to ineffective regulation of self-motion. This hypothesis need to be explicitly tested with behavioral measures such as those used in previous studies on healthy volunteers measuring response times to visual motions coherent or incoherent with gravity [39,58,71] or measuring the anchoring of balance control in response to gravity [72].

In the same study, visual cortex activity (V1, V2, V3) increased during the vertical vs. horizontal comparison in proportion to the severity of dizziness handicap [49]. This effect might be related to heightened visual dependency, but this possibility was not explicitly tested, which could be done by measuring visual dependence in study participants, for example, using the Rod and Disk test [23,32].

#### 3.3.2. Increases in Local Activity

Popp et al. found increased brain responses to the perception of moving dots (inducing motion-after-effect) in patients with PPV vs. controls using fMRI [48]. In particular, they found that patients with PPV display an increased response in the subgenual cingulate cortex relative to healthy individuals [48] (Figure 3B). The subgenual cingulate cortex (32 sg) is involved in processing negative emotions [73]. However, this response is increased by depression [74], which was likely higher in patients with respect to controls. An fMRI study by von Söhsten Lins et al. [54] directly assessing the brain response of patients with PPPD vs. controls to pictures with negative vs. positive affective value showed increased response to aversive stimuli in a posterior parietal area corresponding to the angular gyrus (PGa) and, contrarily to Popp et al. [48], decreased response in the subgenual cingulate cortex [54]. The posterior parietal cortex is involved in the response to visual threat to self as the relay between visual information and motor cortex to produce defensive movements [75]. However, it also regulates attention [76,77,78]. Thus, patients with PPPD might have responded more than controls to a threat that concerned space processing (possibly related to an escape response or they could have been more attentive to the aversive stimulus than controls. Unfortunately, the sample size of this study was not large enough to control for modestly higher mean levels of state anxiety and depression in patients with PPPD than controls, which could have biased the results. However, this was the only study that recruited a control group of participants who had recovered from acute episodes of vertigo or dizziness without developing PPPD, thus accounting for the potential effects of triggering events.

#### 3.3.3. Decreases in Functional Connectivity

**Figure 4:**
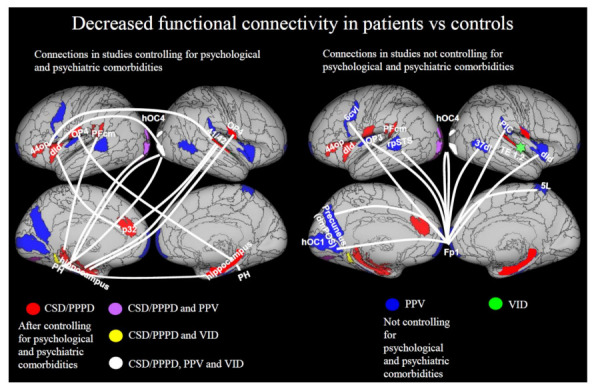
Reduced functional connectivity. In the left panel, we report decreased connectivity after controlling for possible effects of psychiatric comorbidities. After this control, Lee et al. [42] found decreased connectivity in PPPD vs. controls between the left hippocampus and: (1) right central opercular cortex (OP4), (2) bilateral associative cortex (hOC4), (3) left parietal opercular cortex (PFcm). They also showed decreased connectivity between the right hippocampus and: (1) bilateral parahippocampal gyrus, (2) left central opercular cortex (OP4). Indovina et al. [39] found decreased connectivity in CSD/PPPD vs. controls in response to sound-evoked vestibular stimulation between the right superior temporal gyrus (BA 41/42) and: (1) the left hippocampus, (2) the left dId/44 op and (3) left ACC (32p). They also found decreased connectivity between the left dId/44 op and right visual associative cortex (hOC4). Van Ombergen et al. [36] found decreased connectivity of the right central opercular region (TE1.2) with the rest of the brain in patients with VID vs. controls (represented with a star). They also found decreased connectivity between right visual associative cortex (hOC4) and left parahippocampal/temporal fusiform gyrus (PH). In the right panel, we report decreased connectivity in analyses not controlling for possible effects of psychiatric comorbidities. In this analysis, Popp et al. [48] found decreased connectivity in response to visual motion between fpPFC and: (1) right middle temporal gyrus (37dl), (2) bilateral precuneus (right 5 L, left dorso-medial parieto-occipital sulcus = dmPOS), (3) bilateral visual associative cortex (hOC4), (4) right fronto-polar cortex (FP1), (5) left primary visual cortex (hOC1), (6) right PIC, (7) left rpSTS, (8) left parietal operculum (OP3), (9) right dId, (10) left FP1, (11) left 6 cvl. Regions from CSD/PPPD are in red, regions from PPV are in blue, regions from VID are in green. Regions in common between CSD/PPPD and VID are in yellow; regions in common between PPPD and PPV are in purple; regions in common between CSD/PPPD, PPV and VID are in white. Regions from the Anatomy atlas [52] and Brainnetome atlas [53] are overlapped onto the Conte69 inflated brain in workbench viewer [63].

Patients with PPPD and its predecessors showed functional connectivity alterations in many areas of the multimodal-vestibular network [36,39,42,43,44,48]. In analyses controlling for psychological and psychiatric factors [39,42], a decrease of connectivity was evident between areas of the multimodal vestibular network, in particular posterior perisylvian regions, anterior insula and frontal operculum, and limbic areas such as the hippocampus and the anterior cingulate cortex, but also extrastriate areas such as hOC4 (Figure 4 left panel). In studies not controlling for psychological factors, decreased connectivity was identified between many visual and vestibular regions and the frontal polar cortex [48], and between the central insular region and the rest of the brain [36] (Figure 4, right panel). The prominent role of the frontopolar cortex in mood disorders and emotion regulation is well known, suggesting that psychological or psychiatric factors may have had an important influence on these results [79].

Spontaneous functional activity during resting state was also decreased in the right precuneus and cuneus in patients with PPPD relative to controls unmatched for psychological or psychiatric variables in two studies [43,44].

#### 3.3.4. Increase in Functional Connectivity

**Figure 5:**
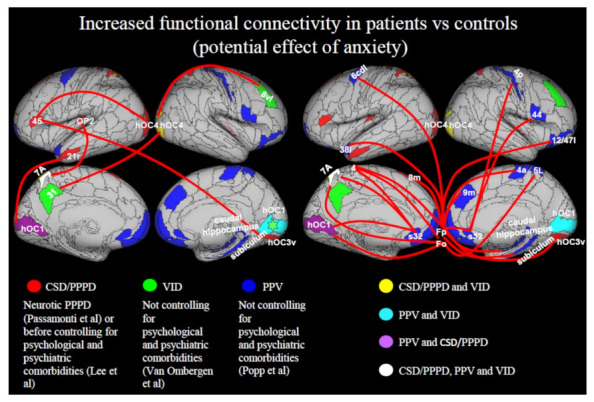
Increased functional connectivity in patients vs. controls (potential effect of anxiety). Significant results are an effect of neuroticism in patients with PPPD (Passamonti et al. [47]), or derive only from analyses in which psychiatric comorbidities were not controlled for. Left Panel: Passamonti et al. [47] considered the effect of neuroticism after matching the control group for neuroticism and found increased connectivity in neurotic patients in response to vertical vs. horizontal stimulation between left BA45 and: (1) right ventral hOC3 (hOC3v), (2) left hOC4. Before controlling for psychiatric comorbidities, Lee et al. [42] (in the seed-based analysis) found increased connectivity between left OP2 and left superior parietal lobe (SPL, 7A) and between left V1 (hOC1) and left temporal pole (rostral BA21 = 21r). Van Ombergen et al. [42] found increased connectivity of right primary visual cortex (hOC1) with the rest of the brain in patients with VID vs. controls. They also found increased functional connectivity between the right visual associative cortex (hOC4) and: (1) right middle frontal gyrus (8vl), (2) left precuneus (31). Right panel: Popp et al. [48] found increased connectivity in response to visual motion stimuli between Fronto-polar (Fp) cortex and: (1) right orbital gyrus (lateral BA 12/47 = 12/47l), (2) right subiculum, (3) bilateral primary motor area (BA4, 4a), (4) left medial temporal pole (lateral BA38 = 38l), (5) right s32, (6) right anterior cingulate cortex (9m), (7) left pre-SMA (BA 8 m), (8) right V1 (hOC1), (9) left premotor cortex (caudal dorso-lateral BA6 = 6cdl), (10) right hippocampus; between orbito-frontal cortex (Fo) and: (1) left V1 (hOC1), (2) bilateral SPL (5L, 7A), left primary motor area (BA4); between subgenual anterior cingulate cortex (s32) and: (1) right inferior frontal gyrus (BA44), (2) left V1 (hOC1), (3) bilateral primary motor area (4, 4p), (4) left 7 A. Connections between regions have been split into 2 panels to avoid overcrowding of the image. Regions from CSD/PPPD are in red, regions from PPV are in blue, regions from VID are in green. Regions in common between CSD/PPPD and VID are in yellow; regions in common between CSD/PPPD and PPV are in purple; regions in common between PPV and VID are in cyan; regions in common between CSD/PPPD, PPV and VID are in white. Regions from the Anatomy atlas [52] and Brainnetome atlas [53] are overlapped onto the Conte69 inflated brain in workbench viewer [63].

Increased functional connectivity was found in patients with PPPD as an effect of neuroticism, a personality trait linked to a high tendency to worry and experience negative emotions [47] and in studies not controlling for psychological or psychiatric factors [44,48]. Thus, it may have been related to higher levels of state or trait anxiety in patients with PPPD (Figure 5). In particular, Passamonti et al. [47] considered how PPPD was influenced by individual differences in neuroticism by contrasting neural correlates of neuroticism in patients with PPPD vs. controls matched by neuroticism scores. They found that in patients with PPPD who scored high in neuroticism, the prefrontal cortex (BA45) was hyperactive, while connectivity between the inferior frontal gyrus (BA 45) and associative visual areas (right hOC3v, left hOC4) increased in response to vertical vs. horizontal self-motion simulation (Figure 5). This may have reflected increased top-down attention in neurotic patients toward the vertical visual context. Popp et al. reported increased functional connectivity between prefrontal areas (fronto-polar, orbito-frontal) and motor, premotor, and superior parietal cortices in patients with PPV [48] (Figure 5). In the study by Lee et al. [42], an increase in frontal and occipital network response was reported, but when controlling for psychiatric comorbidities the significance of these results vanished [42]. Li et al. [44] found enhanced functional connectivity between the sensorimotor network (SMN), which includes the supplementary motor area, sensorimotor cortex, and secondary somatosensory cortex [80], and the occipital visual network (oVN). These results may relate to abnormal postural control exerted by patients with PPPD, due to excessive vigilance about maintaining balance and over reliance on visual rather than vestibular stimuli [27,28,29,30,31].

## 4. Future Directions

### 4.1. The Role of Psychiatric Conditions in PPPD

Quantifying the effects of trait and state anxiety and depression is essential for understanding the PPPD pathophysiology. Anxiety exacerbates space and motion discomfort, and this is likely to be mediated by the shared neural pathways that process visuo-vestibular and emotional stimuli [81]. As observed in anxious individuals, an inability to extinguish conditioned fear responses [82] may play a role in the pathophysiology of PPPD, although this remains to be tested directly. Holle et al. studied habituation to pain in PPPD and found that patients with PPPD displayed, relative to controls, lower pain habituation in response to repeated pain stimuli [83]. This supports the theory that the etiopathogenesis of PPPD may involve an increased response to painful or distressing stimuli, not confined to vestibular/visual inputs but also extending to other sensory modalities [83]. However, that study controlled for depression but not for anxiety or body vigilance, two psychological factors that are more relevant than depression for PPPD and habituation to noxious stimuli. von Söhsten Lins et al. addressed the question of whether patients with PPPD respond more strongly than controls to generic aversive visual stimuli that do not possess provocative features specific to PPPD using the International Aversive Picture System [54]. Between-group comparisons showed that the angular gyrus responded more to negatively than positively valenced stimuli in patients with PPPD compared to controls, whereas within-group analysis found an unexpectedly absent amygdalar response in patients with PPPD, despite them having higher mean ratings of anxiety than comparison patients who had recovered from acute vestibular syndromes. Unfortunately, the study was underpowered for testing whether these differences were due to the psychological state of patients with PPPD or other factors such as differences in arousal or attention, which may affect responses to aversive stimuli in the parietal cortex.

It is also worth noting that increased connectivity in various brain regions (Figure 5) was reported only in studies that did not control for psychiatric comorbidities or trait anxiety [36,42,48]. Therefore, the results described in the literature thus far may be partially confounded by anxiety or related psychopathological conditions [84]. Future studies will have to disentangle how psychiatric variables influence the risk of developing PPPD and modulate key symptoms.

An investigation by Passamonti et al. was the only study that addressed the effect of neurotic personality, i.e., trait anxiety, in PPPD by studying patients with PPPD and a control group matched for neuroticism [47]. They suggested that variable levels of neuroticism in PPPD may modulate brain responses to stimuli that are relevant for PPPD pathogenesis. In healthy volunteers, neuroticism increases the hOC1 activity and connectivity with the prefrontal cortex (left dorsal BA 44) in response to vestibular stimulation [85], suggesting that neuroticism may have an effect on determining prefrontal-visual network hyperactivity [85,86]. One could speculate that the observed increased connectivity of the prefrontal-visual network related to neuroticism may promote visual dependence in patients with PPPD potentially as an attempt to overcoming abnormalities in the visual-vestibular network, or because visual inputs can provide information on potential threats while they are at a distance whereas vestibular and somatosensory inputs cannot.

### 4.2. Integration of Neuroimaging with Mathematical Models of Functional Disorders

The growing number of neuroimaging studies in PPPD hold the promise of revealing more details about its underlying pathophysiologic mechanisms. However, studies published to date highlight several problems that must be addressed in the design of future neuroimaging investigations. First, comparison groups may be needed to identify changes in brain structure and function associated specifically with PPPD versus the effects of its various precipitants and co-existing illnesses. This would entail enrolment of patients who developed PPPD after various types of precipitants (e.g., peripheral vestibulopathies, vestibular migraine, anxiety disorders, mild traumatic brain injury, etc.) and comparable groups of patients having those disorders but no vestibular symptoms, as well as healthy controls. This would allow comparisons between healthy controls and non-PPPD patients to account for the effects of precipitating or comorbid illnesses, while comparisons between non-PPPD and PPPD groups elucidate changes due to PPPD itself. Psychological variables must be measured, and study cohort sizes must be large enough to allow the general linear models of imaging data to include them as covariates, as in the study of Lee, et al. [42]. Longitudinal studies are also needed to understand whether the structural and functional changes in PPPD occur as a consequence of developing the disorder itself or whether they are pre-existing.

Recent advances in machine learning will offer increasing opportunities to discriminate between patients with PPPD and controls, as also shown by Lee and colleagues [42]. Novel hierarchical Bayesian formulations of psychosomatic disorders also may be applied to PPPD to mechanistically describe its symptoms [87]. When combined with data from physiological and neuroimaging investigations, this ought to enable neuro-otologic research and clinical vestibular medicine to abandon the hierarchical dichotomy of ‘organic’ vs. ‘non-organic’ thinking that has emphasized structural lesions as the primary causes of vestibular disorders, relegated psychological variables to a lesser status, and all but ignored altered functioning of body and brain as principal mechanisms of illness. We envisage new theoretical frameworks that focus on interactions among structural integrity, functional status, and psychological process including Bayesian models that would take into account prior expectations about sensory perception and predictions of motion. One theory is that all functional disorders are created by attentional and prediction-driven processes that lead to failure in perceptual inference [87]. In particular, functional motor and sensory symptoms are considered to arise from abnormal attentional bias toward prior symptom-related expectations. This is in line with the classical explanations for PPPD in which excessive attention to postural control and visual stimuli (visual dependence) may be the cause of dizziness symptoms. This model would be in accordance with increased activity and connectivity within attentional, visuo-vestibular and motor networks, as is reported in some cases in PPPD and its predecessors (Figure 2B; Figure 3B; Figure 5).

On the other hand, in PPPD, the internal model of gravity may be altered [49] and this could be tested with Bayesian approaches that model the discrepancy between ‘top-down’ predictions of gravity inputs vs. ‘bottom-up’ sensory evidence [87].

### 4.3. Concluding Remarks

In summary, several cortical visuo-vestibular areas undergo anatomical and functional changes in PPPD. Decreases in structural parameters are observed in the multi-modal vestibular cortex in patients with PPPD relative to controls. Functional alterations in prefrontal and emotional regulatory areas and regions involved in visual-vestibular processing have also been repeatedly described in PPPD. Together, these neuroimaging findings may explain core symptoms of PPPD such as postural unsteadiness and visually induced dizziness. The hypothesis considering anxiety-related personality traits as predicting factors for PPPD remains to be explored; however, the importance of accounting for psychiatric comorbidities in PPPD is not disputed. Increased connectivity within visuo-motor systems may reflect abnormal postural control and balance strategies based on prioritizing safety in the aftermath of precipitating illnesses to the extent that it paradoxically inhibits the return to optimally stable posture and fluid locomotion. Neuroimaging studies conducted thus far in PPPD have also revealed shortcomings in statistical power and measurement of confounds and covariates that must be addressed in the design of future investigations. New avenues that combine improved neuroimaging study designs with computational methods such as Bayesian models hold even more promise for translating emerging research data into novel tools to improve the diagnosis and therapy of PPPD.

## Figures and Tables

**Table 1:** Neuroimaging studies in patients with PPPD or related disorders.

	Number of Patients	Diagnosis	Trigger ^a^	Depression Accounted for	Anxiety Accounted for	Protocol ^b^
Wurthmann et al. [51]	42	PPPD	13 vestibular 29 non-vestibular (unknown)	no	no	grey matter volume
Popp et al. [48]	37	PPV	non-vestibular (unknown)	no	no	grey matter volume and cortical thickness, visual motion stimulation during fMRI
Nigro et al., 2019 [46] Indovina et al., 2015 [39] Riccelli et al. [49] Passamonti et al. [47]	18	CSD/PPPD	vestibular	yes	yes	cortical folding sound evoked vestibular stimulation during fMRI visually evoked self-motion simulation during fMRI
Van Ombergen et al. [36]	10	VID	9 vestibular 1 non-vestibular (unknown)	no	no	Resting state fMRI
Lee et al. [42]	38	PPPD	9 vestibular 4 migraine 1 anxiety 14 non-vestibular (unknown)	yes	yes	Resting state fMRI
Li et al., 2020 [43,44]	12	PPPD	not available	no	no	Resting state fMRI
Na et al. [45]	25	PPPD	6 vestibular 6 emotional stress 2 presyncope 2 sleep deprivation 1 mild head trauma	no	no	Resting state during SPECT
Roberts et al. [50]	17	VID	vestibular	no	no	Caloric vestibular and visual motion stimulation during fMRI
von Söhsten Lins, et al. [54]	16	PPPD	Patients with PPPD (N = 16) 5 psychiatric disorder 4 metabolic disorder 4 peripheral vestibular 2 dysautonomia 1 other Patients recovered from acute vestibular syndromes (N = 16) 8 metabolic disorder 5 peripheral vestibular 2 substance-related 1 other	no	no	Stationary images with positive, neutral, and negative emotional valence during fMRI. **Note. Comparison patients had recovered fully from their acute vestibular syndromes by the time they underwent neuroimaging.**

Studies on PPPD and conditions antecedent to the PPPD definition, such as PPV, VID and CSD. ^a^ Vestibular trigger indicates vestibular neuritis or BPPV. ^b^ The last column describes the neuroimaging technique and protocol used (i.e., resting state or stimulus-triggered).

## Data Availability

Not applicable.
